# Focal Diastematomyelia in an Adult: A Case Report

**DOI:** 10.7759/cureus.26081

**Published:** 2022-06-19

**Authors:** Wahab A Gbadamosi, Amit Daftari, Sandor Szilagyi

**Affiliations:** 1 Diagnostic Radiology, Medical Center of Trinity, Trinity, USA

**Keywords:** bilateral lower extremities weakness, hemicord, spinal cord abnormality, split cord in adult, diastematomyelia

## Abstract

Diastematomyelia (DSM) is a rare congenital malformation that splits the spinal cord longitudinally into two by either cartilage, bone, or fibrous septum. There are multiple case reports of DSM in the pediatric population, but only a few cases of DSM in adult patients have been reported in the literature. This case report describes a middle-aged female patient who presented to the hospital with progressive worsening bilateral proximal lower extremity weakness. A neurological exam was significant for effort-dependent bilateral proximal lower extremity weakness. In addition, magnetic resonance imaging was consistent with an incidental finding of a focal structural-developmental anomaly of diastematomyelia at the distal conus medullaris of the spinal cord vertebral level L2-L3. Following no acute imaging or laboratory abnormalities, the patient was treated with pain management, physical therapy, and outpatient follow-up care. Even though there are multiple differential diagnoses of bilateral lower extremity weakness in adult patients, diastematomyelia malformation is rarely diagnosed in this age group. Therefore, knowledge of this rare congenital anomaly in adult patients should be familiar to interpreting radiologists and treating clinicians.

## Introduction

The embryogenic development of the spinal cord depends on cellular stimuli, genetics, and multiple environmental factors [1-2]. During this embryogenesis journey, many errors can lead to numerous pathological consequences. Diastematomyelia (DSM) is one of these consequences that can emerge. DSM is a type of split cord malformation (SCM), which refers to a category of spinal dysraphism when there is a longitudinal split in the spinal cord by either cartilage, bone, or fibrous septum [1-3]. There are multiple case reports of DSM in the pediatric population, but only a few have been reported in the adult population. Since there are numerous differential diagnoses of bilateral lower extremity weakness in adult patients, congenital spinal cord malformations are rarely diagnosed in this population. Hence, this paper will discuss a case report of an adult patient with a focal DSM who presented with bilateral lower extremity weakness.

## Case presentation

This case presents a middle-aged adult female patient with a two-week history of progressive worsening bilateral proximal lower extremity weakness, which was associated with ambulation difficulty. The patient did not endorse trauma, back pain, bladder-bowel incontinence, saddle anesthesia, extremity paresthesia, headache, fever, or recent viral illness. The patient’s medical and surgical history was non-contributory. There was no documented drug allergy. The patient was not on any home medications and denied tobacco, alcohol, or recreational drug use. Family history was significant for cardiovascular disease.

On physical examination, the temperature was 36.8°C, heart rate was 90 beats per minute, blood pressure was 132/72 mmHg, respiratory rate was 18 beats per minute, and oxygen saturation was 98% on room air. The patient was alert and oriented to self, time, place, and situation. The pupils were anicteric, round, and reactive to light. The mucosal membranes were moist, and the neck was supple. The lungs were clear to auscultation bilaterally. The heart sounds were normal, without gallops, murmurs, or pericardial rub. The abdomen was soft, non-distended, and had normal bowel sounds, no guarding, or costovertebral region tenderness. The lower back demonstrated a painless range of motion. The neurology exam showed intact cranial nerve 2-12, normal speech, bilateral proximal muscle groups power of grade 4/5, a negative Romberg test, normal heel to shin test, and no lower extremity sensory or motor deficits.

The comprehensive metabolic panel (CMP) and complete blood counts (CBC) were within normal limits. Lumbar puncture shows clear cerebrospinal fluid, with normal limits of cell counts and protein. Antibodies for acute viral etiologies, the venereal disease research laboratory test (VDRL), the rapid plasma reagin (RPR), and Lyme disease were negative. Vitamin B12, folic acid, and B6 were all within normal limits. Additionally, urinalysis, toxicology, and TSH were unremarkable.

**Figure 1:**
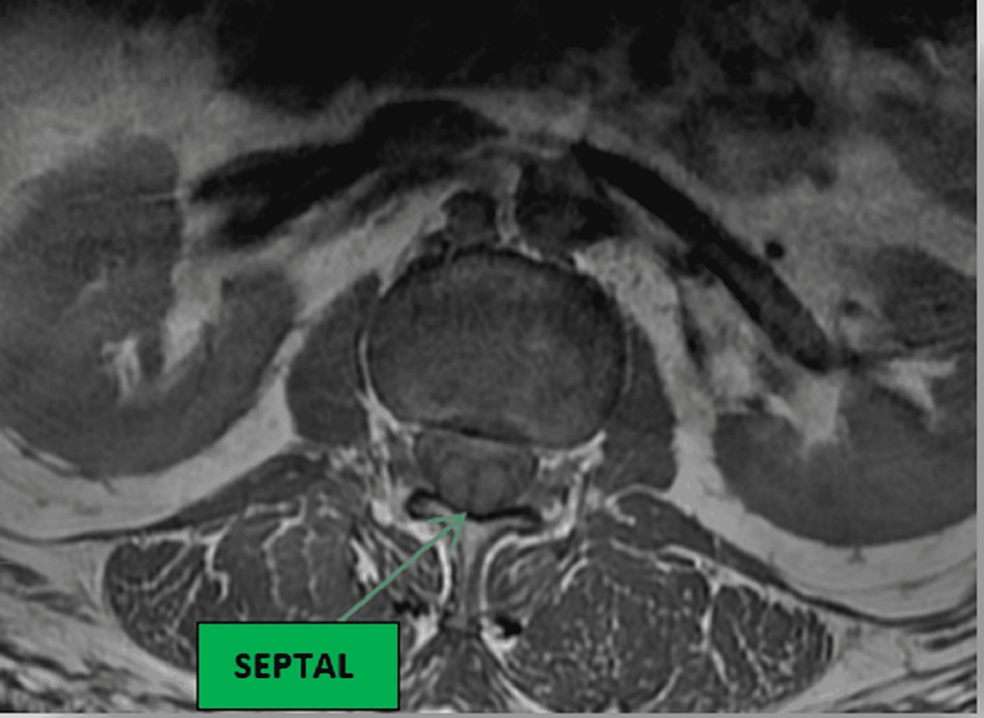
T1-weighted axial lumbar MRI showing a split cord septal at the level of L2-L3.

**Figure 2:**
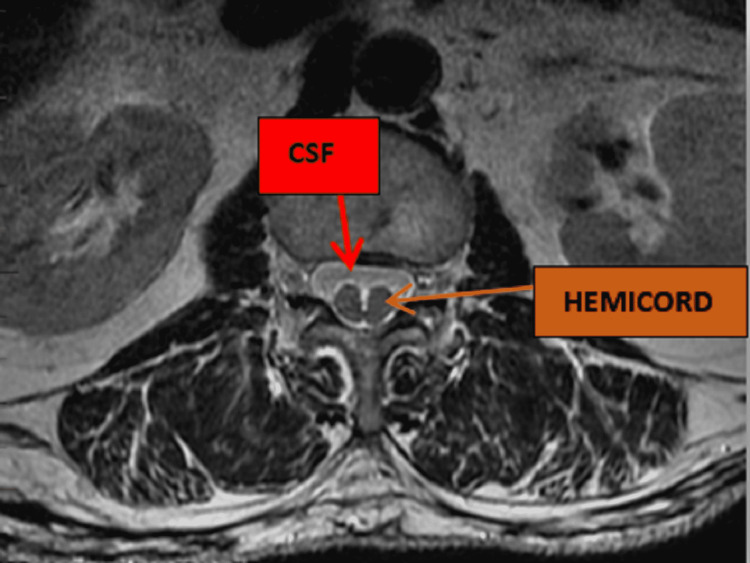
T2-weighted axial lumbar MRI showing two separate hemicords at the level L2-L3.

The brain computed tomographic (CT) and magnetic resonance imaging (MRI) showed no acute etiology. Further imaging workup with MRI of the lumbar spine revealed a mild L5-S1 bilateral foraminal stenosis with an incidental finding of split spinal cord malformation at the L2-L3 vertebral level, which was consistent with a congenital spinal cord anomaly of DSM (Figures 1-2). The rest of the spinal cord, filum terminale, and cauda equina were within the normal limit.

Neurology, neurosurgery, physical therapy, and pain management specialists were consulted throughout the hospital course. Concerns for acute inflammatory demyelinating polyneuropathy (AIDP), acute transverse myelitis, chronic inflammatory demyelinating polyneuropathy (CIDP), acute cerebral vascular accident, or infectious etiologies were low after extensive workup. Given DSM was a congenital variant of a spinal cord and without any acute cord abnormalities from patient symptoms after extensive workup, surgical intervention was not recommended. The patient participated in physical therapy and pain management while in the hospital. An outpatient follow-up at a neurology facility for an electromyogram (EMG) and nerve conduction study (NCS) was scheduled for further neuropathy evaluation.

## Discussion

Diastematomyelia was initially used in 1837 by Dr. Oliver, which he described as a rare congenital malformation of the spinal cord [1]. Despite DSM being rare, it is the most common abnormality associated with congenital scoliosis in pediatric patients [4]. According to Shen et al., the prevalence of DSM in a group of 251 congenital scoliosis in pediatric patients was 16% [4]. The overall prevalence of DSM in the general adult population is unknown [5]. Therefore, most cases are discovered in the pediatric population and are rarely present in adults [6]. Generally, DSM is incidentally found in older patients when medical imaging tests are performed for a different medical diagnosis. In our patient, DSM was an incidental finding unknown to the patient until presenting to the hospital for worsening bilateral lower extremity weakness and difficulty with ambulation.

The embryogenesis of DSM has not been understood fully; however, one of the proposed mechanisms is the neurenteric canal, which transiently connects the yolk sac to the amniotic cavity [1,7]. This medium is believed to have an essential role in forming DSM via duplication of the neural enteric canal [1,7].

DSM is classified broadly into two types. Type one is the classic DSM characterized by the duplication of an embryogenic dural tube with hemicords and type two is a single embryogenic dural tube with a divided spinal cord [8-10]. Furthermore, DSM congenital malformation has been associated with intra- and extra-dural findings. These include hypertrichosis, hemangioma, vertebral scoliosis, hydromyelia, syringomyelia, and spina bifida [8-10]. These findings are primarily associated with type one DSM, while type two lacks most related features. Since DSM is rarely present in adult patients, the non-specific symptoms associated with this disorder can lead to numerous diagnoses of radiculopathy syndromes [6]. Therefore, DSM should be familiar to clinicians when adult patients present with weakness in the lower extremities or myelopathy changes [6,11].

DSM is mainly diagnosed with medical imaging, and the thoracolumbar spine region accounts for 50% of all cases [12]. Radiographs may not reveal the presence of DSM in a patient with a fibrous septum or cartilaginous division; however, a combination of CT with myelogram or MRI can reveal the actual state of the spinal cord [6]. Therefore, the ideal radiological assessment of DSM is best with MRI, which will show a symmetrical cord split with a dural sac that identifies type one, or a unilateral dura sac with hemicords, which identifies type two. The spine MRI can also assess for any other surrounding structure anomalies. In our patient, the lumbar MRI did visualize the septum with a focal symmetrical hemicord and double dural sac at the lumbar spine level L2-L3 without any associated cord abnormality as described in Figures 1-2.

Patients with DSM malformation can benefit from either surgical or medical management. Surgical intervention may be considered in a patient with progressive neurological impairment. In contrast, non-symptomatic patients may benefit from pain management, rehabilitation centers, muscle function test, nerve stimulators, and physical therapies [6].

## Conclusions

DSM is a unique congenital anomaly that is well known in the pediatric population but not in adults, and our case report highlights this rare spinal disorder in adults. Diagnosis relies mainly on magnetic resonance imaging in this patient age group. Patients with severe neurological impairment may benefit from surgical intervention. Therefore, clinicians caring for adult neurological symptoms should be familiar with this rare congenital spinal cord malformation to prevent unnecessary imaging and laboratory workups in adult patients. Future works on the effect of DSM on adult patients may provide the morbidity impact DSM has on adult patients.
